# Giant vulvar inclusion cyst in a 50-year-old female: a case report

**DOI:** 10.1097/RC9.0000000000000856

**Published:** 2026-09-08

**Authors:** Haitham Abdalla Ali Ismail, Abdulrahman Mohammed Abdulrahman Abouh, Ahmed Abdalla Agab Eldour

**Affiliations:** a Department of Obstetrics and Gynaecology, Faculty of Medicine and Health Sciences, University of Kordofan, El Obeid, Sudan; b Department of Surgery, Faculty of Medicine and Health Sciences, University of Kordofan, El Obeid, Sudan; c Department of Surgery, El Obeid Teaching Hospital, El Obeid, Sudan; d Department of Pathology, Faculty of Medicine and Health Sciences, University of Kordofan, El Obeid, Sudan

**Keywords:** case report, female genital mutilation, giant vulvar mass, postmenopausal presentation, surgical excision, vulvar epidermal inclusion cyst

## Abstract

**Introduction and importance:**

Epidermal inclusion cysts (EICs) of the vulva are common benign lesions that are usually small and asymptomatic. However, giant vulvar EICs are extremely rare, particularly in women presenting after menopause, where vulvar malignancy must always be excluded. In countries with a high prevalence of female genital mutilation (FGM), vulvar EICs may occur secondary to chronic vulvar trauma.

**Case presentation:**

We report the case of a 50-year-old married multiparous woman (para 3) with a 16-year history of progressive right vulvar swelling that became painful during the preceding 2 months after menopause. The swelling had spontaneously ruptured three times previously with discharge of whitish keratinous material before recurring again. Examination revealed a large right-sided lower vulvar cystic mass measuring approximately 15 × 11 cm, with smooth overlying skin and no inguinal lymphadenopathy. Ultrasound demonstrated a well-circumscribed avascular hypoechoic subcutaneous lesion measuring 16 × 12 cm. The patient underwent complete surgical excision under spinal anaesthesia in the lithotomy position through a longitudinal vulvar incision. Histopathological examination confirmed a fibrous-walled EIC lined by keratinized stratified squamous epithelium and filled with lamellate keratin debris without evidence of malignancy. The postoperative course was uneventful, and no recurrence was observed during 6 months of follow-up.

**Clinical discussion:**

Giant vulvar EICs are uncommon and may clinically mimic vulvar malignancy or other benign vulvar tumors. Careful clinical examination, imaging, complete surgical excision, and histopathological assessment are essential for definitive diagnosis and exclusion of malignancy.

**Conclusion:**

This case highlights the importance of considering EICs in the differential diagnosis of giant vulvar masses in women presenting after menopause. Complete surgical excision remains the definitive diagnostic and therapeutic approach.

## Introduction

Vulvar masses encompass a broad spectrum of benign and malignant lesions ranging from cysts and hamartomas to aggressive vulvar neoplasms^[^1,2^]^. Epidermal inclusion cysts (EICs) are among the most common benign vulvar lesions and generally arise following trauma, surgery, chronic inflammation, or implantation of epidermal tissue beneath the dermis^[^3,4^]^. Although most vulvar EICs are small and asymptomatic, giant lesions are exceedingly rare.

HIGHLIGHTS

- Extreme rarity: The presentation of a giant epidermal inclusion cyst (EIC) measuring 16 × 12 cm in the vulva of a 50-year-old postmenopausal woman is an exceptional clinical rarity.
- Diagnostic challenge: The large size and the patient’s age necessitated an urgent and comprehensive differential diagnosis to exclude vulvar malignancy, which is a primary concern in postmenopausal women.
- Malignancy eisk: This case underscores the non-negligible risk of malignant transformation reported in giant EICs, thereby making complete surgical excision mandatory.
- Definitive management: Complete surgical excision provided both a definitive diagnosis (benign EIC confirmed by histopathology) and curative treatment, resulting in an excellent long-term outcome.

In Sudan and other African and Middle Eastern countries, female genital mutilation (FGM) is recognized as an important etiologic factor in vulvar EICs, particularly clitoral inclusion cysts, which may present several years after the initial procedure^[^5^]^.

The evaluation of any enlarging vulvar mass in women presenting after menopause requires careful assessment to exclude vulvar malignancy. Large vulvar masses may mimic malignant tumors clinically because of their size, progressive enlargement, and associated symptoms^[^6^]^.

We report a rare case of a giant vulvar EIC in a 50-year-old woman that originated during the premenopausal period and progressively enlarged and became painful after menopause. This case highlights the diagnostic challenges and surgical management of giant vulvar masses in resource-limited settings.

This case report has been reported in line with the SCARE 2025 guidelines^[^7^]^.

### Patient perspective

While advanced imaging tools were not available for the diagnostic process, the patient expressed gratitude for the prompt diagnosis and surgical treatment she received. She was relieved to learn the reason for her symptoms and satisfied with the care she received throughout the hospitalization and recovery process.

## Case presentation

### Patient presentation

A 50-year-old married multiparous Sudanese woman (para 3) presented with a 16-year history of slowly progressive right vulvar swelling. The swelling initially appeared during the premenopausal period and progressively enlarged over time. During the 2 months following menopause, the swelling became painful and caused discomfort with walking and daily activities.

The patient reported three previous spontaneous ruptures of the same vulvar swelling, associated with the discharge of thick whitish material and followed by recurrence. She also complained of recurrent burning during micturition. There was no history of diabetes mellitus, hypertension, smoking, previous vulvar surgery, sexually transmitted diseases, or drug allergies.

The patient denied significant sexual dysfunction; however, she reported difficulty maintaining local hygiene because of the large size of the mass.

There was no documented history of FGM, although it could not be definitively excluded.

### Clinical evaluation

The patient was hemodynamically stable on admission. Local examination revealed a large right-sided lower vulvar mass measuring approximately 15 × 11 cm and weighing about 250 g. The swelling had a smooth surface with intact overlying skin and was freely mobile, cystic in consistency, non-tender, fluctuant, and non-transilluminant. There was no ulceration, discharge, or palpable inguinal lymphadenopathy (Figs. 1 and 2).

**Figure 1.**
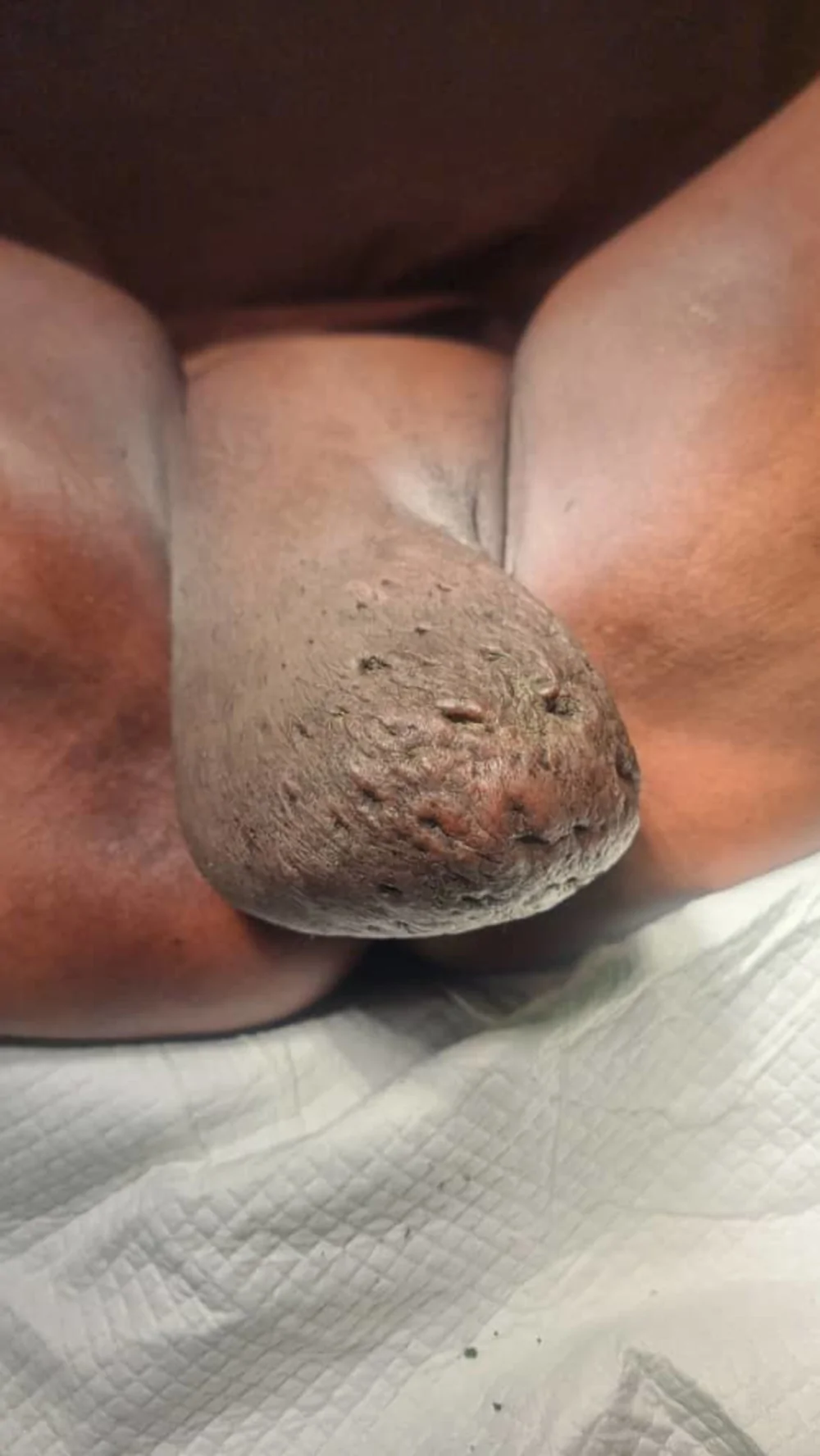
Clinical image, anterior view, showing a large vulvar swelling with a smooth surface and intact overlying skin, measuring approximately 16 × 12 cm, located in the right vulvar region.

**Figure 2.**
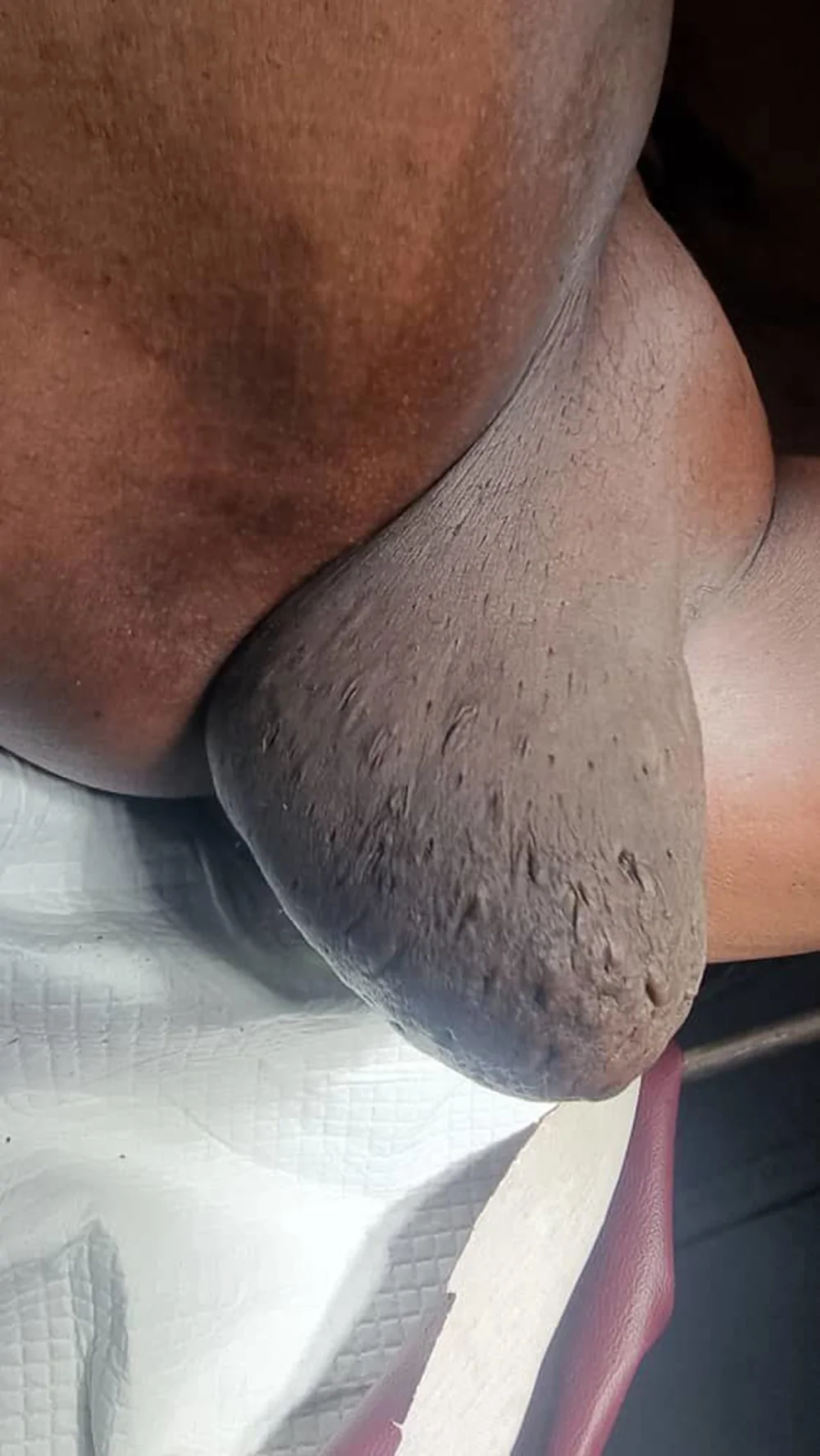
Lateral view of the vulvar mass demonstrates well-defined margins, cystic nature, and free mobility, with no signs of skin ulceration or inguinal lymphadenopathy.

The lesion was not attached to the urethra or vagina, although its large size likely contributed to the patient’s recurrent burning on micturition.

### Diagnostic workup

Soft tissue ultrasonography demonstrated a well-defined oval hypoechoic subcutaneous lesion measuring approximately 16 × 12 cm without internal vascularity, suggestive of a benign cystic lesion.

Magnetic resonance imaging (MRI) was not available in our resource-limited setting.

Routine laboratory investigations were within normal limits.

### Surgical intervention

The patient underwent surgery under spinal anesthesia in the lithotomy position. After standard antiseptic skin preparation and sterile draping, a longitudinal vulvar incision was made over the swelling. Complete en bloc excision of the cyst was successfully performed without rupture of the cyst wall.

The excised specimen weighed approximately 250 g and contained laminated keratinous material on sectioning.

The mass was carefully dissected from adjacent vulvar structures with preservation of surrounding tissues. The wound was irrigated and closed in layers. A urinary catheter was inserted intraoperatively and removed 6 hours after surgery Fig. 3.

**Figure 3.**
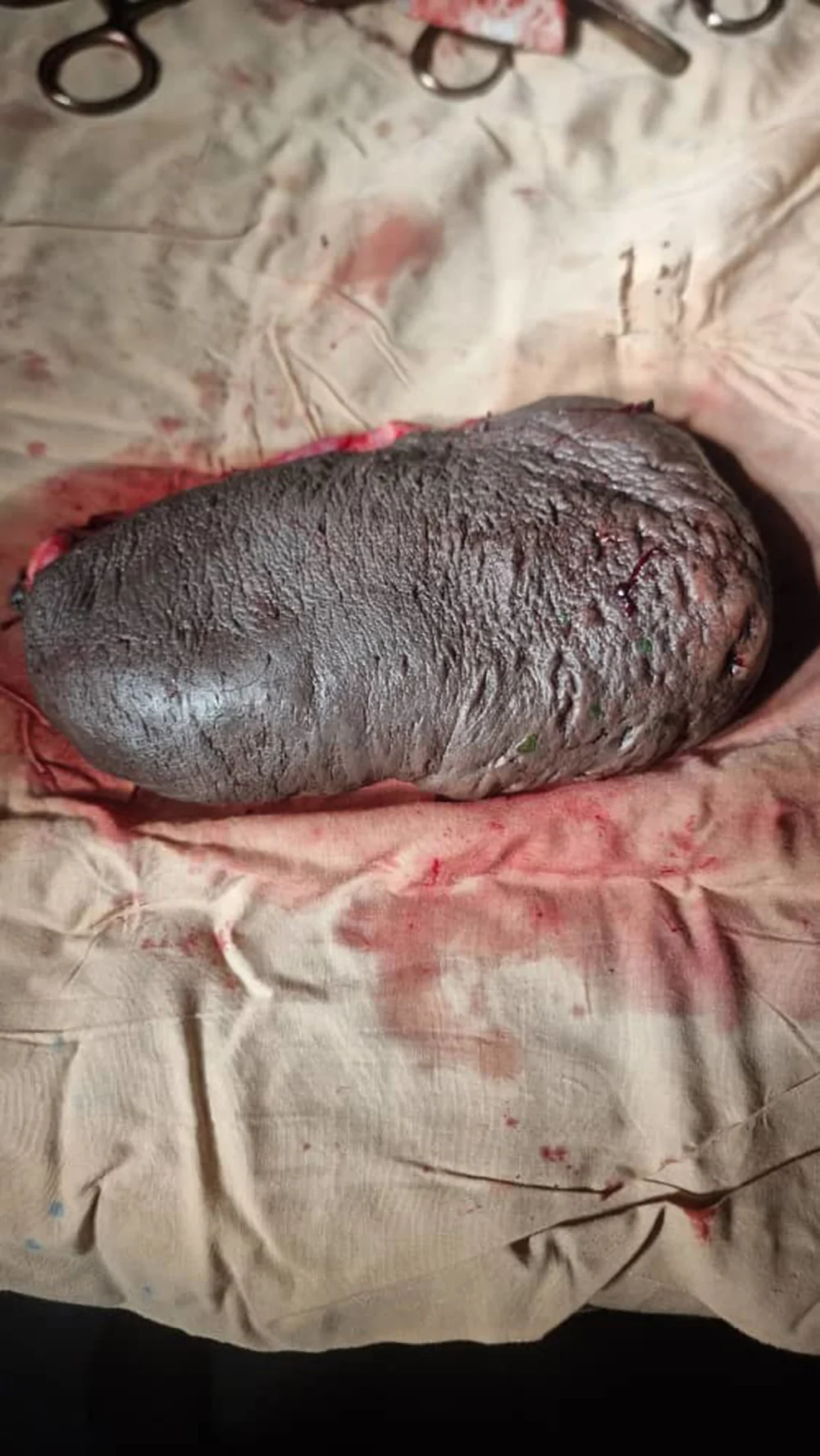
Excised specimen of the giant vulvar epidermal inclusion cyst, displaying an intact fibrous wall and a homogeneous cystic structure prior to histopathological processing.

Histopathological examination revealed a fibrous-walled cyst lined by keratinized stratified squamous epithelium and filled with lamellated keratin debris, without evidence of dysplasia or malignancy.

### Outcomes and follow-up

The postoperative course was uneventful. The patient was discharged on postoperative day 1 in good condition. Sutures were removed on postoperative day ten, and wound healing was satisfactory. Follow-up over 6 months demonstrated no recurrence or postoperative complications Table 1).

**Table 1:** Giant vulvar inclusion cyst in a 50-year-old female: a case report.

Day	Events
1	Onset of vulvar swelling
60	Associated with pain
60	Clinical exam, labs, ultrasound → subcutaneous swelling
61	Excisional biopsy
62	Discharged home on postoperative day 1
71	Stitches removal
85	Follow-up

## Discussion

Giant vulvar EICs are uncommon benign lesions that may clinically resemble vulvar malignancies, particularly in women presenting after menopause. Although EICs are generally small and asymptomatic, long-standing lesions may progressively enlarge over several years before presentation^[^3,4^]^.

In countries where FGM is prevalent, vulvar EICs may develop secondary to chronic vulvar trauma and implantation of squamous epithelium beneath the dermis^[^5^]^. Such lesions may remain asymptomatic for many years before becoming clinically evident.

The differential diagnosis of giant vulvar masses includes Bartholin cysts, lipomas, vulvar fibromatosis, giant vulvar polyps, abscesses, and vulvar malignancies^[^6,8–10^]^. Careful clinical examination and imaging are essential to characterize the lesion and exclude invasive disease.

In our patient, ultrasonography demonstrated a well-circumscribed, avascular, cystic lesion suggestive of a benign process. MRI may provide superior soft tissue characterization and assessment of adjacent structures; however, it was unavailable in our setting.

Complete surgical excision remains the treatment of choice for giant vulvar EICs because it provides both definitive diagnosis and curative management while minimizing recurrence risk. Histopathological examination is mandatory to confirm the diagnosis and exclude malignant transformation, which has rarely been reported in long-standing lesions.

## Conclusion

Giant vulvar EICs are rare benign lesions that may clinically mimic vulvar neoplasms, particularly in women presenting after menopause. Comprehensive clinical evaluation, appropriate imaging, complete surgical excision, and histopathological assessment are essential for accurate diagnosis and successful treatment.

## Data Availability

Data is available at any time.
